# Efficacy of *Cordyceps militaris* Extracts against Some Skin Pathogenic Bacteria and Antioxidant Activity

**DOI:** 10.3390/jof8040327

**Published:** 2022-03-22

**Authors:** Kiratiya Eiamthaworn, Thida Kaewkod, Sakunnee Bovonsombut, Yingmanee Tragoolpua

**Affiliations:** 1 Department of Biology, Faculty of Science, Chiang Mai University, Chiang Mai 50200, Thailand; kiratiya_eiam@cmu.ac.th (K.E.); thida_kaewkod@cmu.ac.th (T.K.); sakunnee.b@cmu.ac.th (S.B.); 2 Graduate School, Chiang Mai University, Chiang Mai 50200, Thailand; 3 Research Center in Bioresource for Agriculture, Industry, and Medicine, Department of Biology, Faculty of Science, Chiang Mai University, Chiang Mai 50200, Thailand

**Keywords:** *Cordyceps militaris*, cordycepin, antibacterial activity, skin pathogenic bacteria, antioxidant activity

## Abstract

*Cordyceps militaris* has been used for treating various diseases, as well as maintaining good overall health. The antibacterial properties of the *C. militaris* fruiting body and substrate, cultured in Chiang Mai (sample A and B) and Chiang Rai (sample C), Thailand, were investigated in this study. The aqueous and ethanolic extracts of *C. militaris* exhibited antibacterial activities against *Staphylococcus aureus*, *Pseudomonas aeruginosa*, *Cutibacterium acnes* and methicillin-resistant *S. aureus* (MRSA) with the MIC/MBC ranging from 3.91 to 31.25 mg/mL. The ethanolic extracts of the fruiting body and substrate from sample B also inhibited all bacterial growth within 2–4 h of treatment. Furthermore, ethanolic extract from sample B showed the highest cordycepin content of 57.42 mg/g extract, whereas the highest adenosine content, 3.78 mg/g extract, was observed in the ethanolic extract from the fruiting body of sample A by HPLC. The ethanolic extracts from sample A also demonstrated the highest antioxidant activity and flavonoid content by 9.50 mg GAE/g extract and 10.59 mg QAE/g extract, respectively. However, the highest phenolic content of 49.04 mg GAE/g extract was found in the aqueous extract of sample A. In addition, the ethanolic extract of sample A at 2 and 4 mg/mL could significantly down-regulate the *mecA* gene expression in MRSA. Our findings reported the potential of *C. militaris* extract as a new substance for the treatment of skin pathogenic bacteria infections and an antioxidant agent.

## 1. Introduction

Skin infections are usually caused by bacteria such as *Staphylococcus aureus*, *Pseudomonas aeruginosa*, *Cutibacterium acnes* and methicillin-resistant *Staphylococcus aureus* (MRSA) [1]. The infections of the skin and soft tissue from skin pathogenic bacteria can stimulate human body responses such as skin inflammation and some skin diseases, including impetigo, furuncles, carbuncles, cellulitis and erysipelas [2,3,4]. The skin infections were treated using antibiotics and synthetic agents, however, the increasing case number of antibiotic resistant bacteria has been recently reported [5]. Furthermore, the excessive use of antibiotics may have adverse effects on health. Thus, the replacement of drugs with natural substances is a good alternative option for the treatment of bacterial infections due to their safety, efficiency, and low toxicity. *Cordyceps militaris* is a parasitic entomogenous fungus that can form a fruiting body using the pupae or larvae of lepidopteron insects [6,7]. *C. militaris* is an edible mushroom and classified in the phylum Ascomycota, class *Ascomycetes*, order *Hypocerales* and family *Clavicipitaceae* [6]. The *C. militaris* has been known as a crude medicinal drug that has proved its usefulness for the maintenance of health and for protection and treatment of various diseases [8]. Cordycepin exhibited a broad spectrum of antimicrobial activities against microorganisms such as *Bacillus cereus, Micrococcus flavus, Escherichia coli, S. aureus, Aspergillus fumigatus* and *Aspergillus niger* [9]. The medical and biotechnological properties of *C. militaris* are due to the presence of many bioactive compounds such as small molecules, secondary metabolites, polysaccharides, proteins, cordycepin, cordymin, cordycepic acid, adenosine, carotenoids, enzymes, organic selenium, ergosterol, sterol, superoxide dismutase (SOD) and multivitamins. Moreover, *C. militaris* and other mushrooms are sources of bioactive peptides possessing anti-hypersensitive, antibiotic, antimicrobial, antioxidant, anti-aging, antibacterial, antifungal, anti-proliferative, anti-inflammatory and ACE inhibitory properties in different human tumor cell lines. Peptides, namely cordymin isolated from *C. militaris* was able to inhibit HIV-1 reverse transcriptase [9,10,11,12,13,14,15].

The aim of this study was to evaluate the inhibitory effects of *C. militaris* extract against pathogenic bacteria causing human skin disease, and to investigate the antioxidant activities and detect the main compounds; cordycepin and adenosine of *C. militaris* extract.

## 2. Materials and Methods

### 2.1. Preparation of C. militaris Extract

Three samples of *C. militaris* cultured in Thailand were selected in this study. Sample A was the fruiting body of the fungi and sample B was the fruiting body mixed with substrate. Both sample A and B were obtained from the Mushroom research and development center, Chiang Mai, Thailand. Moreover, sample C was the fruiting body mixed with substrate that obtained from Chiang Rai, Thailand. The samples were dried and ground into a fine powder before soaking in a solvent with a ratio of 1:10 (*w*/*v*). Ethanolic extract was prepared by soaking *C. militaris* powder in 95% ethanol and shaking at 150 rpm for 72 h to obtain the ethanolic extract. The aqueous extract was prepared by maceration of *C. militaris* powder in distilled water and incubated at 45 °C for 3 h. The insoluble materials were filtered through Whatman No.1 filter paper. Crude extracts of *C. militaris* were concentrated using a rotary evaporator to remove the solvent and dried by lyophilizer. After that, the crude extracts were stored in amber glass bottles at 4 °C until use [16].

### 2.2. Determination of Cordycepin and Adenosine in C. militaris Extracts by High Performance Liquid Chromatography (HPLC)

The amount of cordycepin and adenosine in *C. militaris* extracts was determined using HPLC assay. The sample was filtered through a filter paper containing a pore size of 0.45 µm and 50 µL of filtrate was injected into the HPLC system (Agilent 1200 series). The HPLC condition was determined by reverse phase column (Inertsil ODS-3 4.6 × 150 mm, 5 µm) employing a UV detector of 254 nm. In addition, the HPLC system was controlled with a flow rate of 1.0 mL/min and a running time of 30 min at 30 °C. The mobile phase consisted of water and methanol at ratio of 92:8 (*v*/*v*). The HPLC profile was detected and the content of cordycepin and adenosine in *C. militaris* extracts were calculated from the standard graph of each compound [17].

### 2.3. Determination of Antioxidant Activities of C. militaris Extracts

Antioxidant activity of *C. militaris* extracts was determined by a DPPH radical scavenging method [18]. Various concentrations of *C. militaris* extract were mixed with 1.5 mL of 0.1 mM DPPH solution (Sigma-Aldich, St. Louis, MO, USA). After that, the mixture was incubated at room temperature for 20 min in dark conditions and the mixture was measured by spectrophotometer (Genesys 20, Thermo Scientific, Dreieich, Germany) at an absorbance of 517 nm. Methanol solution was used as a blank control and the absorbance of DPPH solution without extract was used as a control (A control). Meanwhile, the absorbance of DPPH solution mixed with extract was used as sample (A test sample). The antioxidant activities of the extracts were compared to gallic acid as an antioxidant standard. Percentage of DPPH radical inhibition can be calculated using this equation: [(A 517 control-A 517 test sample)/A 517 control] × 100. The antioxidant activity was reported as mg gallic acid equivalent per gram extract (mg GAE/g extract).

### 2.4. Total Phenolic Compound of C. militaris Extracts

*C. militaris* extract (250 µL) was mixed with 125 µL of 50% Folin–Ciocalteu solution (Merck, Darmstadt, Germany), 1.25 mL of DI water and 250 µL of 95% ethanol. Then, the mixture solution was incubated in the dark at room temperature for 5 min. After that, 250 µL of 5% sodium carbonate was added to the mixture and further incubated in the dark at room temperature for 60 min. The absorbance of the mixture was measured at 725 nm [19]. The amount of total phenolic compound was calculated and compared to gallic acid standard curve. Total phenolic content was reported as mg gallic acid equivalent per gram extract (mg GAE/g extract).

### 2.5. Total Flavonoid Compound of C. militaris Extracts

*C. militaris* extract (0.5 mL) was mixed with 0.1 mL of 10% aluminum chloride, 1.5 mL of methanol, 0.1 mL of 1 M of potassium acetate and 2.8 mL of DI water. Then, the mixture was incubated in the dark at room temperature for 30 min [19]. The absorbance of the mixture was measured at 415 nm and the total flavonoid content was calculated by comparing to quercetin standard and was shown as mg quercetin equivalent per gram extract (mg QUE/g extract).

### 2.6. Antibacterial Activity of C. militaris Extracts

The antibacterial activity of *C. militaris* extracts against skin pathogenic bacteria was investigated by agar well diffusion method. Skin pathogenic bacteria including *Pseudomonas aeruginosa* ATCC 27853, *Staphylococcus aureus* ATCC 25923, methicillin resistant *Staphylococcus aureus* (MRSA) and *Cutibacterium acnes* were used in this experiment. All bacterial strains were cultivated in Mueller Hinton Agar (MHA) at 37 °C for 18–24 h except *C. acnes* DMST 14916, cultivated in Brain Heart Infusion Agar (BHIA) at 37 °C for 48–72 h under anaerobic condition. Turbidity of the culture was adjusted to 0.5 McFarland turbidity standard (OD600 nm = 0.08–0.1), which is equal to 1.0 × 10^8^ CFU/mL and swabbed on MHA or BHIA. Wells of 10 mm in diameter were prepared on the agar plate with a sterile cork borer. The *C. militaris* extracts (500 mg/mL) were added into the wells and incubated at 37 °C for 24 h except *C. acnes* that was incubated at 37 °C for 48–72 h. Gentamycin was used as a positive control. The antibacterial activity was determined by measuring the zone of growth inhibition [20].

### 2.7. Determination of Minimal Inhibitory Concentration (MIC) and Minimal Bactericidal Concentration (MBC) of C. militaris Extracts

The minimal inhibitory concentration (MIC) and minimal bactericidal concentration (MBC) were carried out by broth dilution method. The bacterial suspension was adjusted to 0.5 McFarland turbidity standard and transferred to the broth medium containing each concentration of extract after two-fold serial dilution. The mixture in sterile test tube was incubated at 37 °C for 24 h except *C. acnes*, which was incubated at 37 °C for 48–72 h. The MIC value was determined from the lowest concentration of the extract that did not show any bacterial growth when compared to the bacterial growth control. For MBC investigation, the culture tube that appeared invisible for growth of bacteria was streaked on agar plate and incubated at 37 °C for 24 h. The MBC was the lowest concentration that inhibited the bacterial growth by 99.9% [21].

### 2.8. Time Killing Kinetics of C. militaris Extracts

Time killing assay was performed in triplicate. Skin pathogenic bacteria was adjusted to 0.5 McFarland turbidity standard. Then, the bacterial culture was incubated with *C. militaris* extract. After incubation for 0, 2, 4, 6, 12 and 24 h, the number of bacterial colonies were quantified by spread plated technique and the percentage of inhibition was calculated by comparing to the bacterial control [22].

### 2.9. Effect of C. militaris Extracts for Inhibition of Virulence Gene Expression by qRT-PCR

The virulence gene expression of *mecA* in methicillin resistant *S. aureus* (MRSA) was evaluated by qRT-PCR. Bacterial cells were treated with *C. militaris* extract. The bacterial RNA was extracted by NucleoSpin (Macherey-Nagel, Düren, Germany) and reversed to cDNA by ReverTra Ace^®^. The master mix of the reaction contained 4× DN Master Mix and 5× RT Master Mix II, then cDNA was amplified using specific primers by real time RT-PCR. The primer sequences are listed in Table 1 and a transcriptional level of *16s RNA* gene encoding as a ribosomal protein was used for normalization. Cycle threshold (Ct) values of *mecA* gene were calculated from the relative expression by normalization with the expression of the *16s RNA* regulator gene [23,24].

### 2.10. Statistical Analysis

All experiments were operated in three independent treatments. The data was compared and analyzed from treatments and control group. Moreover, the data are also presented as mean ± SD of three independent experiments and revealed significantly different values for each extracts (ANOVA followed by Tukey’s HSD tests, *p* < 0.05).

## 3. Results

### 3.1. C. militaris Extraction

**Figure 1:**
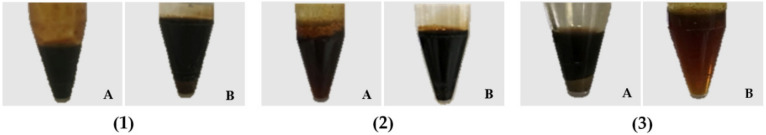
The physical appearance of the ethanolic (**A**) and aqueous (**B**) crude extracts of *C. militaris* from sample A (**1**) *C. militaris* was obtained from the Mushroom research and development center, Chiang Mai, Thailand), sample B (**2**) *C. militaris* fruiting body mixed with substrate was obtained from the Mushroom research and development center, Chiang Mai, Thailand) and sample C (**3**) *C. militaris* fruiting body mixed with substrate was obtained from Chiang Rai, Thailand).

The crude extract of *C. militaris* was extracted by distilled water and 95% ethanol. The percentage yields of aqueous and ethanolic crude extracts are shown in Table 2. The aqueous extract of *C. militaris* sample C showed the highest percentage yield of 19.9%. The percentage yield of aqueous extracts ranged from 12.6–19.9%, while the percentage yield of ethanolic extracts ranged from 3.6–6.2%. The ethanolic extract of *C. militaris* sample A showed the lowest percentage yield at 3.6%. Crude extracts varied in color from light brown to dark brown, depending on the sample and solvent extraction (Figure 1).

### 3.2. Analysis of Cordycepin and Adenosine in C. militaris Extracts by HPLC

**Figure 2:**
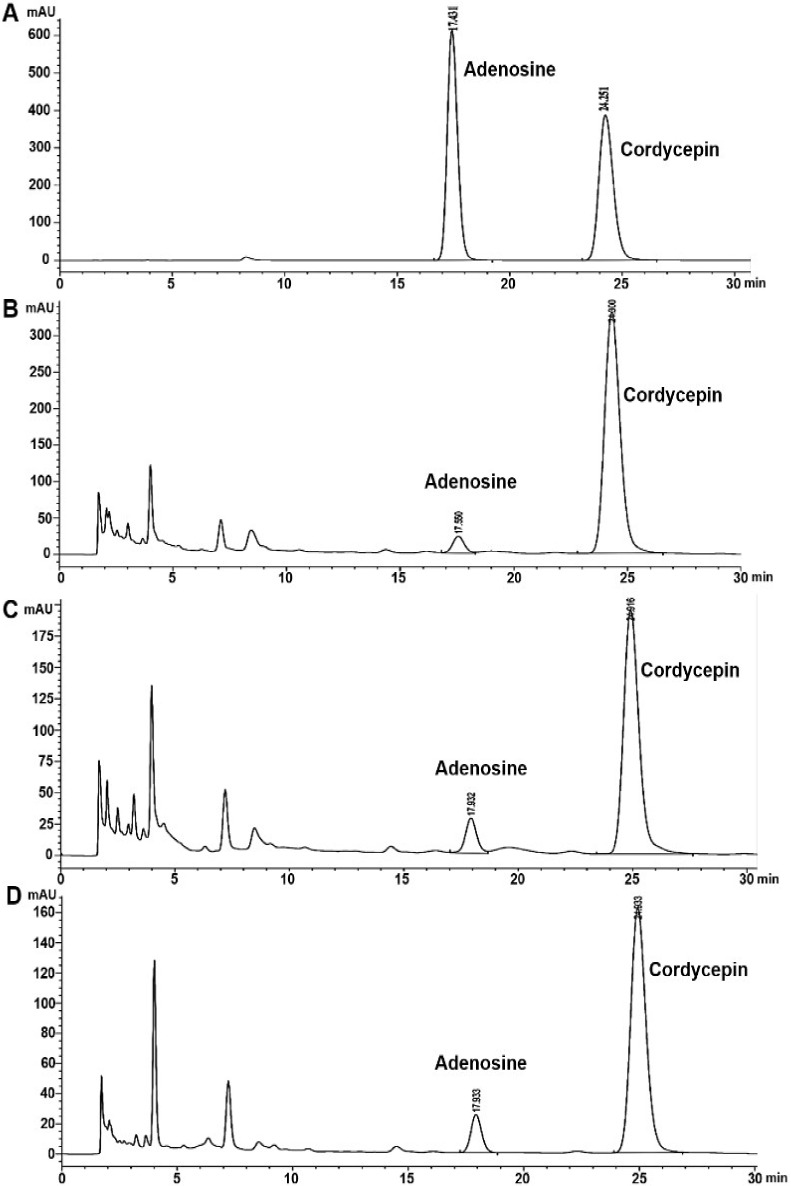
HPLC chromatograms of cordycepin and adenosine founded in the aqueous extracts of *C. militaris* by HPLC method: (**A**) Standard compounds of cordycepin and adenosine; (**B**) Sample A; (**C**) Sample B; (**D**) Sample C.

**Figure 3:**
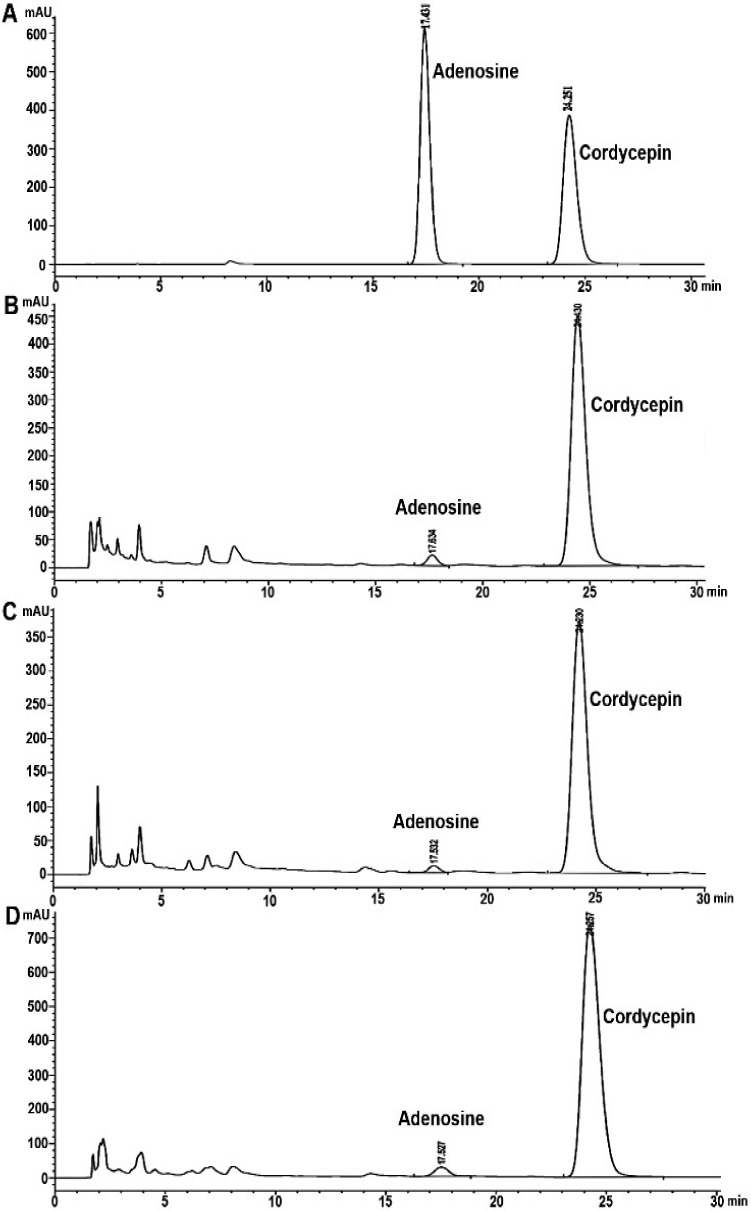
HPLC chromatograms of cordycepin and adenosine founded in the ethanolic extracts *of C. militaris* by HPLC method: (**A**) Standard compounds of cordycepin and adenosine; (**B**) Sample A; (**C**) Sample B; (**D**) Sample C.

The result of HPLC analysis of *C. militaris* extracts was demonstrated in Table 3 and Figure 2 and Figure 3. The highest cordycepin content was shown in both aqueous and ethanolic extracts of sample B with the values of 25.66 ± 0.11 mg/g and 57.42 ± 0.27 mg/g extract. Moreover, the ethanolic extract of all samples showed the cordycepin contents (25.26 ± 0.22–57.42 ± 0.27 mg/g extract) higher than the aqueous extract that expressed the values of 17.15 ± 0.16–25.66 ± 0.11 mg/g extract. In addition, adenosine compound was shown with the highest value of 3.78 ± 0.05 mg/g extract in the ethanolic extract of sample A, and 2.86 ± 0.01 mg/g extract in the aqueous extract of sample A. Moreover, sample B and C extracts from water and ethanol extractions were also performed, an adenosine compound with the values of 2.77 ± 1.25–1.16 ± 0.06 mg/g extract and 0.28 ± 0.01–0.49 ± 0.01 mg/g extract (Table 3 and Figure 2 and Figure 3).

### 3.3. Antioxidant Activities of C. militaris Extracts

In our finding, the ethanolic extract of sample A had the greatest antioxidant activity of 9.50 ± 0.51 mg GAE/g extract, while the aqueous extracts showed the highest scavenging DPPH radical with the value of 9.42 ± 0.75 mg GAE/g extract from the sample B extract (Table 4). Moreover, the aqueous extract of sample A also revealed high antioxidant capacity of 9.12 ± 0.21 mg GAE/g extract. In addition, the aqueous and ethanolic extract of sample C had the lowest antioxidant activity of 3.72 ± 0.26 mg GAE/g extract and 3.76 ± 0.27 mg GAE/g extract (Table 4).

### 3.4. Total Phenolic Compound of C. militaris Extracts

In this study, the result of total phenolic of *C. militaris* extract showed that the aqueous extract of sample A had the highest total phenolic content with the value of 49.04 ± 0.42 mg GAE/g extract, followed by the aqueous extracts of sample B, and C that showed the phenolic content of 45.52 ± 0.56 mg GAE/g extract and 40.03 ± 3.06 mg GAE/g extract, respectively (Table 4). For *C. militaris* ethanolic extract, the sample A also showed the highest total phenolic content with the value of 28.87 ± 1.14 mg GAE/g extract. Subsequently, total phenolic compounds in ethanolic extracts of sample C and B were 24.62 ± 1.34 mg GAE/g extract and 23.71 ± 1.85 mg GAE/g extract (Table 4).

### 3.5. Total Flavonoid Compound of C. militaris Extracts

The present study demonstrated that the aqueous extract of sample C had the highest total flavonoid content of 11.31 ± 0.64 mg QAE/g extract. In addition, the aqueous extract of sample A and B also showed the flavonoid compounds with the total content of 7.45 ± 0.19 and 9.10 ± 0.26 mg QAE/g extract. In addtion, the ethanolic extract of sample A showed the highest total flavonoid content of 10.59 ± 1.98 mg QAE/g extract. However, the ethanolic extract of sample B and C also exhibited the total flavonoid content of 2.85 ± 0.27 and 2.83 ± 0.15 mg QAE/g extract (Table 4).

### 3.6. Antibacterial Activity of C. militaris Extracts by Agar Well Diffusion Method

The results demonstrated that *C. militaris* extracts (500 mg/mL) could inhibit the growth of bacteria causing skin disease. The aqueous extracts of sample A and sample B had inhibitory activity against all four tested bacteria that exhibited the diameters of the inhibition zone ranging from 12.83–20.50 mm. Moreover, inhibitory effects on MRSA, *P. aeruginosa and S. aureus* were shown from an aqueous extract of sample C. In addition, ethanolic extracts of all *C. militaris* samples could inhibit MRSA, *P. aeruginosa and S. aureus* with the diameters of the inhibition zone ranging from 12.17–18.83 mm, except *C. acnes* (Table 5).

### 3.7. Minimal Inhibitory Concentration (MIC) and Minimal Bactericidal Concentration (MBC) of C. militaris Extracts

The results of MIC/MBC values are summarized in Table 6. The ethanolic extracts of samples A and B showed the lowest MIC/MBC values of 3.91 mg/mL against MRSA. Additionally, the aqueous extracts of sample A, B and C showed MRSA inhibition with MIC/MBC values of 15.62, 7.81 and 15.62 mg/mL, respectively.

Moreover, both aqueous and ethanolic extracts of all samples showed antibacterial efficacy against *P. aeruginosa* and *S. aureus* with the MIC/MBC values ranging from 15.6–31.25 mg/mL. Furthermore, the aqueous and ethanolic extracts could inhibit *C. acnes* with MIC/MBC value of 7.81 mg/mL. Hence, the ethanolic and aqueous extracts of *C. militaris* demonstrated antibacterial activity against *S. aureus*, *P. aeruginosa*, *C. acnes* and MRSA.

### 3.8. Time Killing Kinetics of C. militaris Extracts

**Figure 4:**
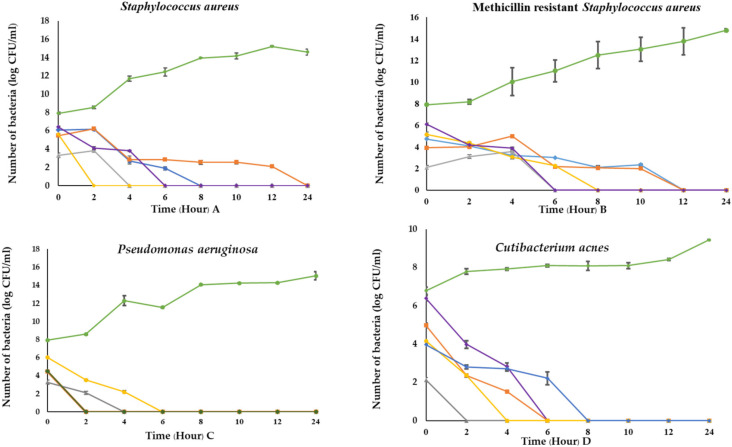
Time-killing curves of bacterial growth of *S. aureus* (**A**), MRSA (**B**), *P. aeruginosa* (**C**) and *C. acnes* (**D**) after treatment with *C. militaris* extracts. Gentamycin was used as a positive control of *S.aureus*, *C. acnes* and *P. aeruginosa*. Doxycycline was used as a positive control of MRSA. The ethanolic extract of sample A (purple line), The aqueous extract of sample A (orange line), The ethanolic extract of sample B (grey line), The aqueous extract of sample B (yellow line), Antibiotic control (blue line), Bacteria control (green line).

The ethanolic extract of sample B at the MIC concentration of 1.96 and 15.62 mg/mL showed the strongest growth inhibition of MRSA and *S. aureus* more than 2-log10 and 3-log10 CFU/mL by completely killing these bacteria within 2 and 4 h, respectively. Moreover, the ethanolic extract of sample B also demonstrated the strongest inhibitory effect at the MIC concentration of 3.91 and 7.81 mg/mL by reducing the growth of more than 2-log10 and 3-log10 CFU/mL when tested against *C. acnes* and *P. aeruginosa*. The viability of *C. acnes* and *P. aeruginosa* was not detected after 2 h of treatment when comparing to untreated bacterial control. Thus, the bacteria decreased rapidly at different times after testing with *C. militaris* extracts (Figure 4).

### 3.9. Effect of C. militaris Extracts on Inhibition of Virulence Gene Expression by qRT-PCR

**Figure 5:**
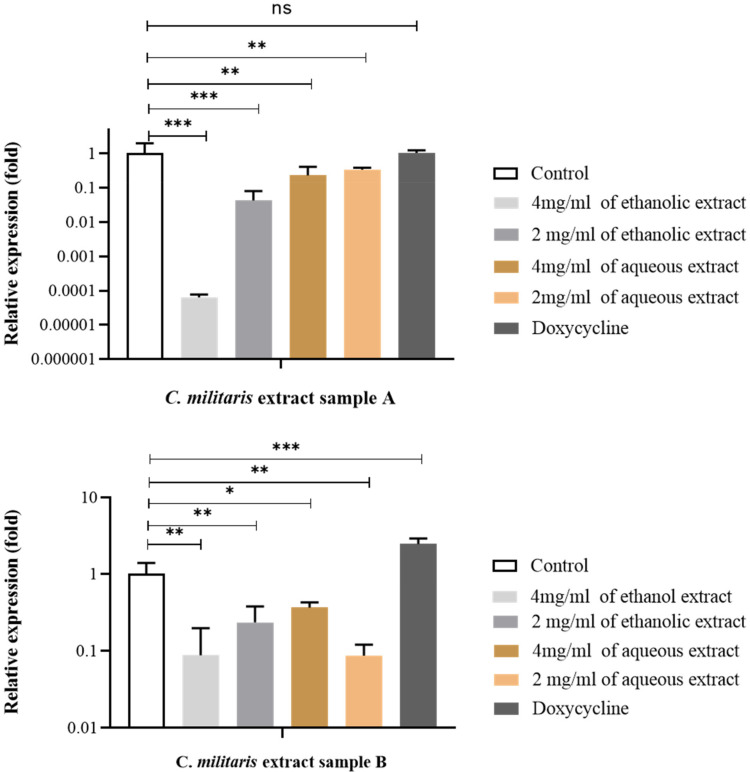
The relative mRNA expression of *mecA* gene on MRSA after treatment with the aqueous and ethanolic extract of *C. militaris* sample (**A**,**B**). The expression of *mecA* gene was compared to the untreated control. Gene expression ratios of MRSA were normalized and compared to the expression of the regulator gene. * indicates *p* < 0.033, ** *p* < 0.002, *** *p* < 0.001, and Not significant (ns).

MRSA contained the *mecA* gene, which conferred the resistance to methicillin drug by reducing the affinity of ß-lactam antibiotics against the MRSA. The mRNA expression of *mecA* gene was studied by qRT-PCR. MRSA was treated with aqueous and ethanolic extract of sample A and B at 37 °C for 24 h. The result showed that the effect of ethanolic extract of sample A at concentration 2 and 4 mg/mL could significantly down-regulate the *mecA* gene. However, the aqueous extract of sample A at a concentration of 2 and 4 mg/mL slightly down-regulated the expression of the *mecA* gene, compared to untreated control (Figure 5).

## 4. Discussion

Bacteria such as staphylococci and streptococci regularly live on the skin and the inner surface of the nose and mouth of humans as normal flora microorganisms. However, some species of pathogenic bacteria can enter into the skin and cause skin infection. Many symptoms such as reddened skin around the injury and pus-filled regions can occur [25]. Most methicillin-resistant *Staphylococcus aureus* (MRSA) causes the majority of community-acquired infections of the skin, accounting for 59% of skin and soft tissue infections [26,27]. Moreover, when people are treated with antibiotic drugs for a long period of time, bacteria can develop resistance to the drugs. To date, the use of natural products against bacteria that cause skin infections is increasing.

In this study, the inhibitory effect of *C. militaris* extract against skin pathogenic bacteria was investigated, as well as the antioxidant activity. Water and ethanol were used as a solvent for the extraction of *C. militaris*. After extraction, the yield of extracts was compared, and the aqueous extract of *C. militaris* exhibited a higher percentage yield than the ethanolic extract. The quantity of the extract is affected by various factors. Different extraction methods, extraction temperature, extraction duration, and solvent polarity could have an impact on the quantity of the extracts [28]. In this study, HPLC revealed that the crude ethanolic extract of *C. militaris* showed the highest cordycepin and adenosine contents. When comparing cordycepin and adenosine content to a previous study of Song et al. [17], the amounts in this study were higher than the previous assay. The different amount of these compounds may depend on many factors; such as solvent ratio, temperature, time and frequency of the extraction [29]. Cordycepin and adenosine were the main bioactive compounds in *C. militaris* that correlated with a wide range of functions including antioxidant and antibacterial properties.

Moreover, previous research has reported that the *C. militaris* contains other bioactive compounds such as polysaccharides, ergosterol peroxide, superoxide dismutase (SOD), fibrinolytic enzyme and carotenoids [30,31]. Recently, *C. militaris* extracts were proved to contain a high source of antioxidants after the discovery of a wide range of bioactive ingredients, such as flavonoids, polyphenols, sterols, peptides and polysaccharides [32]. Many different in vitro assays have been evaluated for antioxidant activity so as to seek out an antioxidant that would be useful for the biological system. In our research, *C. militaris* extracts were evaluated for antioxidant activity by DPPH radical scavenging assay. In addition, the extracts were surveyed for their contents of phenolics and flavonoids, which are responsible for antioxidant activity. In this study, the aqueous extract of *C. militaris* showed the highest scavenging activity on DPPH radicals, relating to a high content of total phenolics and flavonoids. Moreover, phenolic and flavonoid content were also found in *C. militaris* extracts in both the fruiting body and substrate. The antioxidant properties of *C. militaris* extract demonstrated that the scavenging free radicals can protect against oxidative damage to DNA, proteins and other macromolecules. Additionally, *C. militaris* extract had the ability to inhibit the reactive oxygen species (ROS), which can cause cancer and aging diseases [33].

Adenosine and cordycepin isolated from *C. militaris* exhibited several biological activities [34]. Adenosine has benefits for the prevention of ROS-related diseases such as tissue damage, is a therapeutic agent against chronic heart failure, and has anti-inflammation properties [31]. Cordycepin exhibited beneficial effects, such as broad-spectrum antibiotic activity, inhibition of cell proliferation, induction of cell apoptosis, as well as antioxidant activity [35,36].

Therefore, our results revealed that the aqueous extract of *C. militaris* exhibited a higher content of phenolics and flavonoids than the ethanolic extract. The amount of phenolic compound was not the only factor in the consideration of antioxidant activity. Moreover, flavonoid content is also considered, as numerous studies have suggested that flavonoids possess biological properties such as being antioxidant, antiallergenic and anti-inflammatory [37,38]. Our study found that the total flavonoid content in *C. militaris* aqueous extract was higher than the ethanolic extract. However, flavonoids were also found in various extracts when using different solvents for extraction, including water and ethanol [39]. The effects of flavonoids are generally connected to the antioxidant activity of these molecules. Flavonoids take effect via blocking some enzymes that produce reactive oxygen species, or by chelating trace elements implicated in free radical production [40]. Furthermore, the in vitro antioxidant activity has been reported to correlate with the amount of cordycepin and adenosine, which might be responsible for antioxidant performance, as found in previous reports [9,33]. It was concluded that the extracts of *C. militaris* exhibited antioxidant activities through different mechanisms. There were other components responsible for the activity with DPPH, phenol and flavonoids. Other studies reported that active compounds, such as polysaccharides from aqueous extract of *C. militaris,* exhibited antioxidant properties [34,41], although there was little polysaccharide content found in the ethanolic extract [42].

To date, the extracts from fungi are interestingly considered to use for antimicrobial activities. As an important medicinal fungus, *C. militaris* has also been tested for its antimicrobial activities [42]. The results of the investigation revealed that *C. militaris* extracts had varying degrees of antibacterial activity against the microorganisms examined. In our study, the aqueous extract of *C. militaris* exhibited stronger antimicrobial activity than the ethanolic extract by the agar disc diffusion method. However, the MIC and MBC had shown the highest activity from the ethanolic extract as it is directly tested between bacteria and extract, while the agar disc diffusion method might be affected by the diffusion of the extract into the agar. Additionally, antibacterial action was stated to be directly proportional to the cordycepin concentration [43]. Other studies reported that the ethanolic extract of *C. militaris* was more effective than the aqueous extract against *S. aureus* [44,45].

MRSA is reported as a serious problem in health care due to its ability to acquire drug resistant determinants such as erythromycin, clindamycin, ciprofloxacin, tetracycline, gentamycin, etc. [46]. Outbreaks of diseases in a hospital are also caused by MRSA [47]. In this experiment, the *C. militaris* extract from samples A and B were selected to study the effect on the antibiotic-resistant gene expression in MRSA. Specifically, methicillin’s resistance to the mediatory *mecA* gene exposed to methicillin inactivates the high-binding-affinity to penicillin binding proteins (PBPs) that are commonly established in *S. aureus*. However, a PBP2a protein that is encoded by the *mecA* gene, usually present in MRSA, has a low affinity for methicillin. The *C. militaris* extracts inhibited the synthesis of peptidoglycan, allowing MRSA to grow [48]. We hypothesized that the inhibition of the *mecA* gene when combined with *C. militaris* is a possible mechanism against MRSA. In this study, we found that the aqueous and ethanolic extracts of *C. militaris* extract proved significantly effective by the downregulation of the *mecA* gene transcription, that was determined by qRT-PCR. Our study proposed the mechanism action of *C. militaris* on *S. aureus* and revealed an antibacterial action associated with the disruption of cell wall biosynthesis, similar to other known cell walls interfered by antibiotics [49]. Moreover, the phenolic compound could interfere with membrane integration or associated proteins, and interfere with their production of the bacterial membrane [50,51].

## 5. Conclusions

*Cordyceps militaris* is one of the most popular and important mushrooms for its nutraceuticals in Southeast Asia. In this study, the antibacterial and antioxidant properties of the aqueous and ethanolic extracts of *C. militaris* were investigated, as well as the content of phenolics, flavonoids, cordycepin and adenosine. The results showed that skin pathogenic bacteria, including *S. aureus*, *P. aeruginosa*, *C. acnes*, and MRSA, were inhibited by *C. militaris* extracts. In addition, the *C. militaris* extracts exhibited significant antioxidant activity. The aqueous extract had the highest ability to inhibit DPPH radicals. Moreover, phenolic and flavonoid content was also found in *C. militaris* extract. Therefore, the effective antibacterial and antioxidant activities were revealed in the *C. militaris* extracts and correlated with the content of cordycepin and adenosine that was also found in the extracts. Interestingly, from this research, *C. militaris* extracts expressed the novel ability to down-regulate the expression of the *mecA* gene in MRSA. Hence, *C. militaris* extracts can be applicable as supplementary active substances that play a role in health promotion.

## Figures and Tables

**Table 1:** Oligonucleotides used for qRT-PCR.

Genes	Sequence of PCR Primers (5′–3′)
*mecA*	Forward: GGCTATCGTGTCACAATCGTTGACG Reverse: CAAATCCGGTACTGCAGAAC
*16s RNA*	Forward: GAGTTTTAACCTTGCGGCC Reverse: CCAGGTGTAGCGGTGAAAT

**Table 2:** Percentage yield of *C. militaris* crude extracts from different samples.

*C. militaris*	Extraction Yields (%)	
	Aqueous Extract	Ethanolic Extract
Sample A	16.4	3.6
Sample B	12.6	6.2
Sample C	19.9	5.9

**Table 3:** Cordycepin and adenosine contents of *C. militaris* extracts.

*C. militaris*	*C. militaris* Extracts (mg/g Extract)			
	Aqueous Extract		Ethanolic Extract	
	Cordycepin	Adenosine	Cordycepin	Adenosine
Sample A	23.98 ± 0.39 ^b^	2.86 ± 0.01 ^a^	32.08 ± 0.10 ^b^	3.78 ± 0.05 ^a^
Sample B	25.66 ± 0.11 ^a^	2.77 ± 1.25 ^b^	57.42 ± 0.27 ^a^	0.28 ± 0.01 ^c^
Sample C	17.15 ± 0.16 ^c^	1.16 ± 0.06 ^c^	25.26 ± 0.22 ^c^	0.49 ± 0.01 ^b^

^a,b,c^, The data of different superscript letters (^a,b,c^) are revealed significantly different values for each extracts (ANOVA followed by Tukey’s HSD tests, *p* < 0.05).

**Table 4:** Antioxidant activity, total phenolic and flavonoid contents of *C. militaris* extracts.

Aqueous Extracts	Antioxidant Activity (mg GAE/g Extract)	Total Phenolic Content (mg GAE/g Extract)	Total Flavonoid Content (mg QAE/g Extract)
Sample A Sample B Sample C	9.12 ± 0.21 ^a^ 9.42 ± 0.75 ^a^ 3.72 ± 0.26 ^b^	49.04 ± 0.42 ^a^ 45.52 ± 0.56 ^a^ 40.03 ± 3.06 ^b^	7.45 ± 0.19 ^c^ 9.10 ± 0.26 ^b^ 11.31 ± 0.64 ^a^
**Ethanolic Extracts**	**Antioxidant Activity** **(mg GAE/g Extract)**	**Total Phenolic Content** **(mg GAE/g Extract)**	**Total Flavonoid Content** **(mg QAE/g Extract)**
Sample A Sample B Sample C	9.50 ± 0.51 ^a^ 4.53 ± 0.15 ^b^ 3.76 ± 0.27 ^c^	28.87 ± 1.14 ^a^ 23.71 ± 1.85 ^a^ 24.62 ± 1.34 ^a^	10.59 ± 1.98 ^a^ 2.85 ± 0.27 ^b^ 2.83 ± 0.15 ^b^

^a,b,c^, The data of different superscript letters (^a,b,c^) are revealed significantly different values for each extracts (ANOVA followed by Tukey’s HSD tests, *p* < 0.05).

**Table 5:** Inhibitory effect of *C. militaris* extracts on skin pathogenic bacteria by agar well diffusion method.

Aqueous Extracts	Diameter of Inhibition Zone on Tested Bacteria (mm)			
	MRSA	*P. aeruginosa*	*C. acnes*	*S. aureus*
Sample A	20.50 ± 2.00 ^a^	15.50 ± 0.50 ^b^	14.17 ± 0.29 ^b^	18.83 ± 1.26 ^a^
Sample B	19.83 ± 0.76 ^a^	15.33 ± 1.04 ^b^	16.67 ± 1.61 ^b^	18.00 ± 0.87 ^ab^
Sample C	17.00 ± 1.00 ^a^	12.83 ± 0.76 ^b^	0 ^c^	14.17 ± 0.29 ^b^
**Ethanolic Extracts**	**Diameter of Inhibition Zone on Tested Bacteria (mm)**			
	**MRSA**	***P. aeruginosa***	***C. acnes***	***S. aureus***
Sample A	18.83 ± 1.04 ^a^	12.17 ± 0.76 ^c^	0 ^d^	15.17 ± 1.26 ^b^
Sample B	17.17 ± 0.29 ^a^	13.33 ± 0.29 ^b^	0 ^c^	13.33 ± 1.15 ^b^
Sample C	18.00 ± 1.73 ^a^	12.50 ± 0.00 ^b^	0 ^c^	17.67 ± 2.08 ^a^
Gentamycin	0	28.02 ± 0.55	32.70 ± 2.22	27.67 ± 1.63
Doxycycline	25.67± 0.58	ND	ND	ND

^a,b,c,d^ The data of different superscript letters (^a,b,c,d^) are revealed significantly different values for each extracts (ANOVA followed by Tukey’s HSD tests, *p* < 0.05). ND = not determined.

**Table 6:** Minimal inhibitory concentration (MIC) and minimal bactericidal concentration (MBC) values of *C. militaris* extracts against skin pathogenic bacteria.

Extracts	Samples	MIC/MBC of *C. militaris* Extracts (mg/mL)			
		MRSA	*P. aeruginosa*	*C. acnes*	*S. aureus*
Aqueous extracts	A	15.62	15.62	7.81	31.25
	B	7.81	15.62	7.81	31.25
	C	15.62	31.25	7.81	15.62
Ethanolic extracts	A	3.91	15.62	7.81	31.25
	B	3.91	31.25	7.81	15.62
	C	7.81	31.25	7.81	15.62

## Data Availability

Not available.
